# Impacto de la leucemia linfoblástica aguda en el microbioma y lesiones bucales: revisión de alcance

**DOI:** 10.21142/2523-2754-1004-2022-131

**Published:** 2023-12-26

**Authors:** Olga Leticia García Rico, Juan Gerardo Sánchez Medina, Elizabeth Sánchez Becerra, Juan Antonio Cepeda Bravo, Francisco Javier Tejeda Nava, Ana Karenina Rocha Viggiano, Mariana Salgado Bustamante, Saray Aranda Romo

**Affiliations:** 1 Clínica de diagnóstico, Laboratorio de Bioquímica, Microbiología y PatologíaFacultad de Estomatología, Universidad Autónoma de San Luis Potosí. San Luis. Potosí, México. leti095@outlook.com, juan_gerardo@hotmail.es, aelizabeth.sanchezb@gmail.com, sarayaranda@fest.uaslp.mx Universidad Autónoma de San Luís Potosí Clínica de diagnóstico, Laboratorio de Bioquímica, Microbiología y Patología Facultad de Estomatología Universidad Autónoma de San Luis Potosí San Luis. Potosí Mexico leti095@outlook.com juan_gerardo@hotmail.es aelizabeth.sanchezb@gmail.com sarayaranda@fest.uaslp.mx; 2 Departamento de Periodoncia Facultad de Estomatología, Universidad Autónoma de San Luis Potosí. San Luis Potosí, México. antonio.cepeda@uaslp.mx Universidad Autónoma de San Luís Potosí Departamento de Periodoncia Facultad de Estomatología Universidad Autónoma de San Luis Potosí San Luis Potosí Mexico antonio.cepeda@uaslp.mx; 3 Departamento de Imagenología Facultad de Estomatología, Universidad Autónoma de San Luis Potosí, San Luis Potosí, México. francisco.tejeda@uaslp.mx Universidad Autónoma de San Luís Potosí Departamento de Imagenología Facultad de Estomatología Universidad Autónoma de San Luis Potosí San Luis Potosí Mexico francisco.tejeda@uaslp.mx; 4 Laboratorio de epigenética, Facultad de Medicina, Universidad Autónoma de San Luis Potosí. San Luis Potosí, México. anak.biomed@gmail.com, marianasalgadobustamante@gmail.com San Luis Potosí México anak.biomed@gmail.com marianasalgadobustamante@gmail.com

**Keywords:** microbiota, microbioma, leucemia linfoblástica aguda, bacterias orales, lesiones orales, disbiosis, microbiota, microbiome, acute lymphoblastic leukemia, oral bacteria, oral lesions, dysbiosis

## Abstract

**Objetivo:**

Describir el conocimiento existente sobre las alteraciones del microbioma oral (MBO) y la presencia de lesiones orales (LO) en pacientes con leucemia linfoblástica aguda (LLA).

**Materiales y métodos:**

Se realizó una búsqueda electrónica en las bases de datos PubMed, SciELO y Google Académico, y se incluyeron artículos descriptivos, analíticos, observacionales sobre MBO, LO y LLA, se siguieron los criterios PRISMA. Se evaluaron 642, se eliminaron artículos duplicados, reportes de caso y aquellos donde solo reportaron los cambios durante o después del tratamiento quimioterapéutico.

**Resultados:**

Se evaluaron 10 artículos, publicados entre 1997 y 2021, 4 artículos coincidieron que el MBO de pacientes con LLA se encuentra en disbiosis mostrando un aumento significativo de firmicutes (0,1%), bacillus (0,05%) y bacterias oportunistas, como *Moraxella* spp., *Klebsiella* spp. (5,66%), *Pseudomona* spp. (3,77%), *Enterobacter* spp. (1,88%), *Acinetobacter* spp. (1,88%) y *E. coli* (1,08%). las LO más frecuentes reportadas en 5 artículos fueron sangrado gingival espontáneo (3,5%), gingivitis (25%) y úlceras (9,4%).

**Conclusiones:**

La cavidad oral de los pacientes con LLA se encuentra en disbiosis y se identifican LO asociadas. Es necesario establecer estrategias preventivas con un enfoque nicho-ecológico para restablecer el MBO, con la finalidad de disminuir el riesgo de infecciones oportunistas y otras LO durante el tratamiento de quimioterapia.

## Abstract (en)

**Objective:**

To describe the existing knowledge about the alterations of the MBO oral microbiome and the presence of OL Oral Lesions in patients with Acute Lymphoblastic Leukemia ALL.

**Materials and Methods:**

An electronic search was carried out in the PubMed, SciELO, and academic Google databases, and descriptive, analytical, observational articles on MBO, OL, and ALL were included, following the PRISMA criteria. 642 were evaluated, duplicate articles, case reports, and those where only changes were reported during or after chemotherapy treatment were eliminated.

**Results:**

10 articles were evaluated, published between 1997 and 2021, 4 articles agreed that the MBO of patients with ALL is in dysbiosis showing a significant increase in firmicutes 0.1%, bacillus 0.05%, and opportunistic bacteria such as *Moraxella spp*, *Klebsiella spp* 5.66%, *Pseudomonas spp* 3.77%, *Enterobacter spp* 1.88%, *Acinetobacter spp* 1.88% and *E. coli* 1.08%, the most frequent OL reported in 5 articles were spontaneous gingival bleeding 3.5%, gingivitis 25% and ulcers 9.4%.

**Conclusions:**

The oral cavity of patients with ALL is in dysbiosis and associated OL is identified. It is necessary to establish preventive strategies with a niche-ecological approach to restore the MBO, to reduce the risk of opportunistic infections and other OL during chemotherapy treatment.

## INTRODUCCIÓN

La leucemia es un problema de salud pública prioritario por su alta incidencia y tasa de mortalidad. A nivel mundial, se estima que se presentan 20 a 35 casos por cada millón de habitantes al año ^1^, mientras que, en México, durante el 2019, se presentaron entre 5000 y 6000 casos nuevos ^2^. La LLA constituye el 75% de las leucemias en la infancia siendo los niños entre 2 y 5 años la población más afectada ³. La LLA es la consecuencia de la transformación maligna de una célula progenitora linfoide inmadura que tiene la capacidad de expandirse sin control y formar clones de células progenitoras neoplásicas idénticas ^4^^-^^7^. 

Durante el proceso patológico que da origen a la LLA existen cambios en los pacientes a nivel molecular, celular y microbiológico previos a las manifestaciones clínicas. Hay reportes previos de niños recién diagnosticados con LLA sobre los cambios en el microbioma humano (colección de microorganismos que viven en conjunto, que interactúan entre sí, en un entorno; incluyen sus genomas, metabolitos, condiciones ambientales y su interrelación), así como de la microbiota (conjunto de microorganismos que colonizan un nicho determinado) en donde un microorganismo o consorcio prolifera en mayor cantidad alterando las funciones del ecosistema; a este fenómeno se conoce como disbiosis ^8^^,^^9^. Estos cambios se deben a las condiciones de inflamación crónica endógena sistémica y bucal, así como al estrés oxidativo presentes en el hospedero, los cuales tienen un impacto al alterar la diversidad y abundancia de bacterias en el microbioma fecal y oral ^10^^,^^11^. 

En el MBO oral, se ha identificado un aumento de *firmicutes* y *Bacillus*^12^, así como *Streptococcus*, *Staphylococcus* y *Candida*^13^^-^^15^. Las especies bacterianas presentes en la cavidad oral de pacientes con LLA reportadas en estudios previos varían de acuerdo con la población estudiada y la región, por lo que la información que existe a la fecha no es clara sobre cuáles son los microorganismos que se encuentran en disbiosis durante el desarrollo de la LLA. Es indispensable determinar qué bacterias inician el proceso de disbiosis, ya que estas proliferan previamente a la aparición de LO. Conforme avanza la enfermedad, los efectos que tiene la disbiosis en la cavidad oral son más evidentes y se reporta la aparición de una gran variedad de LO, entre las cuales destacan la candidiasis, la caries, el herpes y el sangrado gingival espontáneo ^16^^-^^18^. 

Es necesario recopilar información existente sobre cuáles microorganismos inician la disbiosis y cuáles LO se encuentran en la cavidad oral de pacientes con LLA, con la finalidad de instaurar medidas preventivas para esta población, enfocadas en controlar la proliferación de microorganismos patobiontes y recuperar el equilibrio oral mediante terapias dirigidas a estas especies de microorganismos. Con ello se obtendrán mejores resultados, sobre todo para evitar la aparición de LO. Por tanto, el objetivo de esta revisión de alcance es describir el conocimiento existente sobre las alteraciones del MBO y las LO en pacientes con LLA.

## MATERIALES Y MÉTODOS

### Extracción de los datos 

Se realizó una búsqueda de artículos en bases de datos como PubMed, Google Académico y SciELO, del 15 de febrero hasta abril de 2022, la cual abarcó artículos de 1997 a 2021 en los idiomas inglés, español y portugués. En estos se identificó el MBO presente en pacientes con LLA y LO antes del tratamiento quimioterapéutico. Se siguieron los criterios PRISMA.

### Estrategia de búsqueda

La estrategia de búsqueda en PubMed, Google Académico y SciELO utilizó palabras clave con términos (MeSH) y operadores boleanos en los campos de título y resumen, para así identificar los posibles artículos seleccionados (Tabla 1).

**Tabla 1:** Estrategia de búsqueda artículos en las bases de datos

MeSh	Operador	Base de datos
Microbiota	Not	PubMed
Microbiotae	Comillas	Google Académico
Microbiome	And	SciELO
Microbic	Or	
precursor cell lymphoblastic leukemia lymphoma	*	
leukemia lymphoma	()	
bacterias orales		
oral bacteria, manifestaciones orales		
lesiones orales		
oral manifestations lymphoblastic leukemia indicator		
oral lesions		

### Selección de datos

Los resultados obtenidos mediante la estrategia de búsqueda descrita anteriormente fueron cargados al *software* Covidence para realizar el proceso de selección de los artículos. Para evitar posibles sesgos de selección de los artículos, este proceso fue realizado de forma independiente por SN y CW.

Se eliminaron los artículos duplicados y, posteriormente, se evaluó su elegibilidad mediante el filtrado basado en el resumen y título. Así, se examinaron los títulos y se eliminaron los artículos no relacionados con MBO en pacientes con LLA y LO en pacientes con LLA. En un segundo filtro, basado en el resumen, fueron excluidos los artículos que no cumplieron con los siguientes criterios: artículos que no obtuvieran datos sobre MBO y bacterias orales de pacientes con LLA antes del inicio del tratamiento quimioterapéutico; artículos de casos clínicos; artículos con una muestra reducida de pacientes incluidos; artículos publicados en revistas predatorias (https://predatoryjournals.com/journals/, https://beallslist.weebly.com/, https://guides.library.yale.edu/c.php?g=296124&p); artículos donde los datos reportados de pacientes con LLA fueran durante el tratamiento y donde no se reportaran LO antes del tratamiento quimioterapéutico. 

Con respecto a los criterios de inclusión, fueron seleccionados estudios clínicos realizados en humanos en los que se describió la diversidad y abundancia del MBO en pacientes con LLA; artículos que reportaron la presencia de bacterias orales a nivel de género y especie en LLA; ybartículos epidemiológicos sobre LO en pacientes con LLA. Los artículos estuvieron escritos en inglés, español y portugués.

Para la extracción de la información se optó por utilizar tablas donde se resumieran los datos de importancia para esta revisión con los siguientes datos: título de artículo, nombre del autor, objetivo, población, tipo de muestra y microorganismos presentes / LO presentes. Esto permitió capturar de una mejor forma los hallazgos de cada uno de los estudios incluidos.

## RESULTADOS

**Figura 1:**
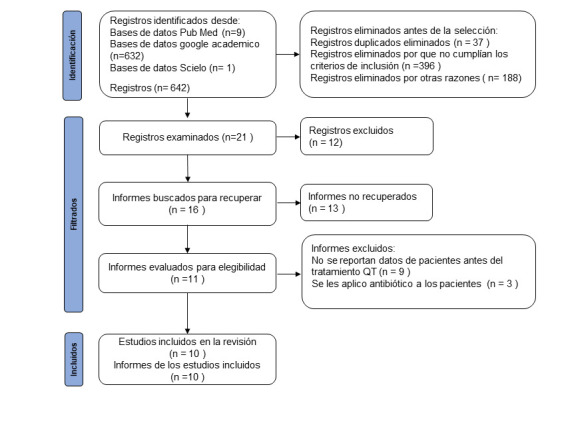
Diagrama PRISMA de los documentos identificados en las bases de datos

De un total de 642 artículos identificados en la búsqueda bibliográfica, se eliminaron 621, (artículos duplicados: 37, eliminados porque no cumplían los criterios de inclusión: 396 y eliminados por otras razones: 188), de los cuales solo 10 artículos fueron incluidos en esta revisión. La estrategia de búsqueda según los criterios prisma se representa en la Figura 1.

**Figura 2:**
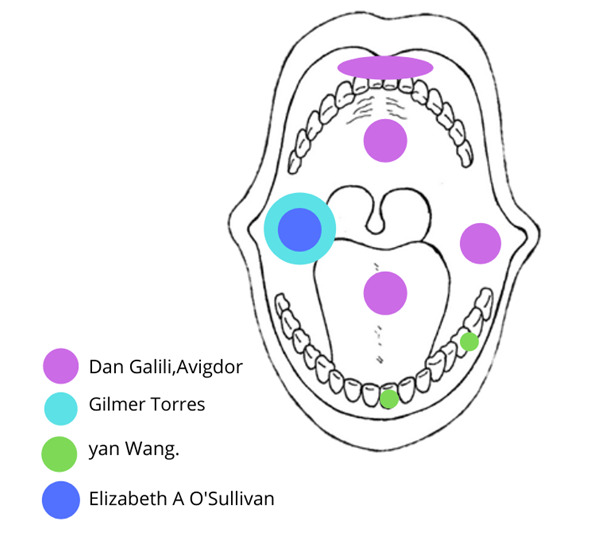
Zonas de donde se obtuvieron las muestras

De acuerdo con la revisión, 4 artículos coincidieron en que el MBO de pacientes con LLA sufre modificaciones significativas que llevan a un estado de disbiosis, a pesar de que la metodología utilizada en cada estudio fue diferente en cuanto a la toma de muestra (saliva, placa dentobacteriana, mucosa bucal) (Fig. 2) y a la técnica utilizada para la identificación de los microorganismos. Tres estudios realizaron cultivos en placas de agar sangre, agar azida, Agar MacConkey, agar manitol salado y agar Sabouroud, y se identificaron los siguientes microorganismos: estreptococos alfa-hemolíticos, estafilococos coagulasa negativos, *Candida albicans* y *Staphylococcus aureus*^19^^,^^20^.

Dos artículos reportaron un incremento de *Streptococcus mutans*^19^^,^^20^; un artículo, incremento de cándida ^19^; otro artículo evaluó los microorganismos entéricos detectados en la flora oral de los pacientes con LLA y las cepas predominantes fueron *Klebsiella*, *Klebsiella pneumoniae*, *Klebsiella* sp., *Klebsiella oxytoca*, *Enterobacter*, *Enterobacter cloacae*, *Enterobacter aerogenes*, *Pseudomonas*, *Pseudomonas aeruginosa*, *Pseudomonas* sp., *Escherichia coli y Citrobacter freundii*^20^^,^^21^. Solo un estudio llevó a cabo técnicas moleculares para la identificación y reportó la taxonomía completa de los microorganismos encontrados. Se identificó filo (Fusobacterias, Firmicutes), clase (Fusobacterias, Bacilii), orden (Lactobacilos, Fusobacterias), familia (Fusobacteriaceae, Comamonadaceae, Carnobacteriaceae, Aerococaceae) y género (*Vellonella*, *Leptotrichia*, *Granulicatella*, *Comamonas*, *Abiotrophia*). Se dieron a conocer los linajes taxonómicos y se reportó que hay mayor predominio de Fusobacterium, bacilos, lactobacilos, Aerococcaceae, y se concluye que existe un incremento en la familia Estreptocaceae, sin ser significativo ^22^. Adicionalmente, un estudio evaluó la tasa de secreción salival de los pacientes con LLA con recién diagnóstico y durante las fases del tratamiento ^20^. La información complementaria sobre el MBO de pacientes con LLA se resumen en la Tabla 2.

**Tabla 2:** Microorganismos (MO) identificados en la cavidad oral de pacientes con leucemia linfoblástica aguda

Titulo	Autores	Objetivo	Tipo de estudio	País de estudio	Población de muestra	Tipo de muestra	Resultados MO	Conclusión
Cambios en el microbiota oral durante la quimioterapia citotóxica en niños tratados por LLA	O'Sullivan *et al*.	Observar los cambios en la flora oral en el grupo de estudio	Observacional prospectivo	Reino Unido	34 pacientes con leucemia aguda infantil (30 con LLA)	Cavidad oral y saliva	S. mutans 173 UFC	Las bacterias presentes en la LLA son *S. mutans*
Tasa de secreción salival, células de levadura y candidiasis oral en pacientes con LLA	Wahlin *et al*.	Determinar la tasa de secreción salival estimulada en pacientes hospitalizados con LA durante periodos de tratamiento con fármacos citotóxicos en dosis mielosupresoras	Observacional prospectivo	Suecia	29 pacientes con LLA durante dos períodos	Saliva	14 pacientes positivos a Candida	Los pacientes con LLA son positivos a *Candida*
Gram-negative enteric bacteria in the oral cavity of leukemia patients	Galili *et al*.	Examinar si existe una correlación entre las infecciones orales con bacterias entéricas, la leucopenia y las úlceras orales en pacientes adultos con LLA.	Observacional	Israel	16 pacientes sanos y 16 pacientes con LLA	Hisopo estéril de la mucosa de la encía, el paladar, la lengua y las mejillas	Las bacterias predominantes fueron cepas de Klebsiella (*Klebsiella pneumoniae*, 29,5%) Klebsiella sp., 12,2% *Enterobacter cloacae*, 15,7%; *Enterobacter aerogenes*, 3,1%), *Pseudomonas aeruginosa* (13,5%); *Pseudomonas* sp. (2,1%), *Escherichia coli* (10,5 %) y *Citrobacter freundii* (8,4%)	Se cultivaron microorganismos entéricos en 2948 muestras de siendo mayor en el grupo de pacientes con LLA (p<0,05)
Bacterias orales en pacientes con LLA	Torres *et al*.	Establecer la diversidad ecológica bucal, destacando los microorganismos oportunistas en pacientes con LLA	Longitudinal	Perú	106 niños tratados en el Instituto de Enfermedades Neoplásicas de Perú de enero a diciembre de 2001	Muestras de cavidad oral	*E.coli* 1,06% *Pseudomona* 3,77% *Enterobacter* 1,88% *Acinetobacter* 1,88% *Klebsiella* 5.66% *Morxella* 10,37% *Candida* no *albicans* 1,88% *Candida albicans* 25,47% *S. coagulasa* 42,45% *S. aureus* 12,26 *S*. alfa hemolítico 72,64%	Los microorganismos más frecuentes en 106 pacientes con LLA fueron el Estreptococo alfa-hemolítico, Estafilococo coagulasa negativo, *Candida albicans* y el *Staphylococcus aureus*; considerados como patógenos residentes
Oral Microbiota Distinguishes Acute Lymphoblastic Leukemia Pediatric Hosts from Healthy Populations	Wang *et al*.	Conocer la Riqueza y diversidad de la microbiota oral en los niños con LLA	observacional	China occidental	Grupo de pacientes a los que se les acababa de diagnosticar LLA en el Departamento de Hematología y Oncología Pediátricas del Segundo Hospital Universitario de China Occidental, Universidad de Sichuan	Placa dentobacteriana	Firmicutes (p = 0,001) Phylum,Bacilos (p = 0,001) Lactobacillales (p = 0,001), Aerococcaceae (p = 0,001) Firmicutes (p = 0,001) Phylum,Bacilos (p = 0,001) Lactobacillales (p = 0,001) Carnobacteriaceae (p = 0,002) Fusobacterias (p = 0,003) Fusobacterias (p = 0,001)	La microbiota oral de pacientes con LLA se encuentra en disbiosis caracterizada por una reducción de la diversidad y abundancia, alteraciones de bacterias asociadas a infecciones oportunistas sistémicas. Se resalta la importancia de la interacción húesped-microbiota relacionada a complicaciones por infección en esta población.

Con respecto a las LO, 5 artículos que fueron seleccionados coincidieron en que las LO presentes en los pacientes con LLA que más predominan fueron gingivitis, úlceras, ganglios linfáticos palpables y sangrado gingival espontáneo ^16^^-^^18^^,^^22^; esto ocurre por la trombocitopenia, neutropenia o la función comprometida de los granulocitos. Un estudio realizó una comparación de pacientes recién diagnosticados con LLA y población sana en la cual se observa que el 37% de los niños con LLA presentan un sangrado gingival espontáneo y el 9% de la población sana. La palidez de mucosa se presenta en un 31%, mientras que en la población sana no se observa palidez de las mucosas ^17^. Otro artículo dio a conocer las LO en pacientes con LLA con recién diagnóstico y sus etapas del tratamiento; por lo tanto, las LO suelen ser signos o síntomas frecuentes en pacientes con leucemia no diagnosticada ^23^. Los datos sobre la presencia de LO en pacientes con LLA se encuentran en la Tabla 3.

**Tabla 3:** Lesiones presentes en pacientes con leucemia linfoblástica aguda

Titulo	Autores	Objetivo	Tipo de estudio	País	Población de muestra	Exploración de tejidos blandos	Resultados LB	Resumen
Alteraciones bucodentales en niños con LLA bajo tratamiento con quimioterapia	Murrieta-Pruneda *et al*.	Determinar la prevalencia de patologías bucales en pacientes pediátricos con (LLA) con y sin tratamiento QT.	Transversal, observacional y analítico	México	103 pacientes pediátricos con diagnóstico de LLA en el Hospital Infantil de México “Federico Gómez” de la Ciudad de México.	Se exploraron: labios, carrillos, mucosa yugular, piso de boca, paladar, orofaringe y lengua. Se aplicaron los índices epidemiológicos para caries dental: CPOD y ceod (dientes cariados, perdidos, obturados.	Mucositis:40% Candidiasis:4% Gingivitis: 46% Úlceras: 13%	Las manifestaciones bucales de las alteraciones hematológicas como la LLA son frecuentes, y pueden ser manifestaciones primarias de la enfermedad.
Patologías orales como indicadores diagnósticos en leucemia	Stafford *et al*.	Evaluar las patologías orales como indicadores diagnósticos en leucemia	Descriptivo, retrospectivo	Australia	500 pacientes con LLA		Sangrado 30% Periodontitis 5% Linfadenopatia70% Faringitis 10%	Las manifestaciones bucales de las alteraciones hematológicas como la LLA son frecuentes.
Prevalence of oral lesions in and dental needs of patients with newly diagnosed acute leukemia	Watson *et al*.	Evaluar la frecuencia de signos orales y evolución del estado bucal en pacientes con LLA	Descriptivo, retrospectivo	Canadá	Doscientos setenta y seis pacientes	Exploración de cavidad oral	Sangrado 28% Úlceras 27% Inflamación gingival 7,7%	Las manifestaciones bucales de las alteraciones hematológicas como la LLA son frecuentes, así como úlceras e inflamación gingival.
Condiciones de salud bucodental en niños menores de catorce años con leucemia linfoblástica aguda, antes del tratamiento de quimioterapia en el Instituto del Cáncer (SOLCA), Cuenca	Parra *et al*.	Establecer las condiciones de salud bucodental y las características sociodemográficas de los niños con LLA menores de 14 años que acuden al Instituto del Cáncer de Cuenca (SOLCA) y compararlos con los niños sanos que asisten a la clínica dOdontopediatria de la facultad de odontología de cuenca	Analítico de corte transversal	Ecuador	64 niños en tres años, 32 niños con LLA y 32 niños sanos.	Exploración de la cavidad oral	Equimosis25 % Úlcera 9,4% Palidez de la mucosa 31% Sangrado espontáneo Generalizado 3,3% Petequias 46,9%	Los niños con LLA presentaron lesiones estomatológicas patológicas en mayor porcentaje que los niños sanos.

## DISCUSIÓN

El MBO de los pacientes con LLA se encuentra en disbiosis previo al tratamiento de quimioterapia; esto debido a cambios inflamatorios, estrés oxidativo y a la proliferación maligna de linfocitos en la médula ósea que se infiltran en los tejidos linfoides periféricos, incluido el tejido linfoide de la mucosa oral, lo que provoca un deterioro de la inmunidad celular y humoral del huésped, así como la pérdida de la tolerancia inmunológica y aumento de bacterias patobiontes. Estas bacterias colonizan los diferentes nichos de la cavidad oral y producen un desequilibrio en el MBO. En condiciones de salud, la composición de la microbiota previene la colonización o sobre crecimiento de patógenos, interactúa con el sistema inmunológico y mejora la función de barrera contra la contaminación bacteriana del torrente sanguíneo, estos microorganismos pertenecen a los filos Firmicutes, Bacteroidetes, Proteobacteria, Actinobacteria, Spirochaetes y Fusobacteria. Sin embargo, en los sujetos con LLA ^12^ se demostró la existencia de un desequilibrio estructural en el MBO que se caracteriza por una diversidad reducida de microorganismos como son *Comamonas* y *Leptotrichia*^21^, así como por la colonización de bacterias oportunistas, entre las cuales se encuentran *Moraxella* spp., *Klebsiella* spp., *Pseudomona* spp., *Enterobacter* spp., *Acinetobacter* spp. y *E. coli*, aunque otros estudios demostraron que los cambios del MBO en pacientes con LLA son producidos por microorganismos comensales propios del nicho, los cuales pertenecen a cocos grampositivos del filio Firmicutes ^20^^,^^21^.

En la cavidad oral, la disbiosis se manifiesta clínicamente con enfermedades de los tejidos duros y blandos. De los artículos revisados, 5 indican que los pacientes con LLA tienen una mayor prevalencia de LO, entre las cuales se encuentran las úlceras y la inflamación gingival ^17^^,^^18^. Con respecto a la etiología, se ha asociado un incremento de *C. ochracea*, *C. sputigena* o *C. gingivalis* con las úlceras orales ^22^ y el infiltrado leucémico, así como a la acumulación de placa dentobacteriana con la inflamación gingival. Otras manifestaciones orales se presentan por la trombocitopenia, lo que da origen a sangrado gingival y petequias. Estas manifestaciones orales pueden presentarse previas al diagnóstico de LLA y es importante su reconocimiento por profesionales de la salud (odontólogo y médico), para el diagnóstico y tratamiento oportuno mediante la referencia al departamento de hematología oncológica. 

Es importante que el odontólogo establezca medidas preventivas en estos pacientes con un enfoque nicho-ecológico para restablecer el equilibrio del MBO en pacientes con diagnóstico reciente de LLA, ya que ha quedado claro el papel que juega el MBO en la aparición de LO. Este abordaje evitaría la aparición de infecciones oportunistas como candidiasis o herpes intraoral, así como úlceras asociadas con citomegalovirus, las cuales se presentan con una prevalencia del 80% ^25^ durante el tratamiento de quimioterapia, al igual que la mucositis (98%) y la caries (37,3%) ^26^. Reportes previos muestran que el 40% de los pacientes con tratamiento quimioterapéutico presenta mucositis en sus diferentes grados ^27^. Se recomienda, en esta población, el uso de probióticos de las cepas *Biffidobacterium longum*, *Lactobacillus acidophilus*, *Biffidobacterium* (250 a 750 mg diarios durante 5-7 días), *Biffidobacterium infantis* y *Saccharomyces boulardii*, previo al tratamiento de quimioterapia, pues esto reduce en un 68% el riesgo de mucositis si se administra en cantidades adecuadas. Los tratamientos varían mucho en cuanto a dosis y duración, pero, en general, se administran durante 7 días hasta 4 semanas ^26^^,^^28^. El odontólogo debe estar informado sobre la disbiosis que sufre el microbioma oral en pacientes con LLA y definir los protocolos para la atención de estos pacientes antes del tratamiento quimioterapéutico, a fin de evitar efectos adversos de los fármacos en la cavidad oral y mejorar las condiciones orales y sistémicas del huésped.

## CONCLUSIÓN

La cavidad oral de los pacientes con LLA se encuentra en disbiosis y se identifican LO asociadas. Es necesario establecer estrategias preventivas con un enfoque nicho-ecológico para restablecer el MBO, con la finalidad de disminuir el riesgo de infecciones oportunistas y otras LO durante el tratamiento de quimioterapia.

## Fuentes de financiamiento:

La presente investigación no contó con fuentes de financiamiento
